# The Effect of Vitamin D Supplementation on Blood Lipids in Patients with Polycystic Ovary Syndrome: A Meta-Analysis of Randomized Controlled Trials

**DOI:** 10.1155/2021/8849688

**Published:** 2021-01-30

**Authors:** Hong Gao, YanTao Li, WenNan Yan, Fei Gao

**Affiliations:** ^1^Department of Endocrinology, First Hospital of Shanxi Medical University, No. 85 Jiefang South Road, 030000 Taiyuan, Shanxi Province, China; ^2^Department of Obstetrics and Gynecology, First Hospital of Shanxi Medical University, No. 85 Jiefang South Road, 030000 Taiyuan, Shanxi Province, China

## Abstract

**Purpose:**

Studies have found that vitamin D supplementation may improve blood lipids in patients with polycystic ovary syndrome, but the results are controversial, so this study will further analyze the effect of vitamin D supplementation on blood lipids in patients with polycystic ovary syndrome.

**Methods:**

PubMed, Embase, Cochrane Library, CNKI, and Wanfang databases were searched up to May 2020, to identify randomized controlled trials of the effect of vitamin D supplementation on blood lipids in patients with polycystic ovary syndrome. The Cochrane risk of bias tool was applied to assess the risk of bias, and RevMan5.3 software was used for statistical analysis.

**Results:**

Ten studies were included in this study, including 543 subjects. The results of the meta-analysis showed that, compared with placebo, vitamin D supplementation can significantly reduce total cholesterol level (WMD = –11.32, 95% CI = [–14.51, –8.41], *P* < 0.00001), low-density lipoprotein cholesterol level (WMD = –4.83, 95% CI = [–7.52, –2.14], *P*=0.0004), and triglyceride level (WMD = –8.23, 95% CI = [–13.08, –3.38], *P*=0.0009, but the effect on high-density lipoprotein cholesterol level is not statistically significant (WMD = –0.32, 95%CI = [–1.24, 0.60], *P*=0.50).

**Conclusion:**

Vitamin *D* supplementation can significantly reduce total cholesterol, low-density lipoprotein cholesterol, and triglycerides in patients with polycystic ovary syndrome. However, it has no significant effect on high-density lipoprotein cholesterol.

## 1. Introduction

Polycystic ovary syndrome (PCOS) is the most prevalent endocrine condition in women of the reproductive age. The syndrome is characterized by hyperandrogenism, irregular menses, and polycystic ovaries when other etiologies are excluded [1]. It is a multifactorial syndrome presenting with obesity, insulin resistance, dyslipidemia, and other metabolic abnormalities [2]. It was estimated that 70% of newly diagnosed women with PCOS had borderline or high lipid levels [3] including increased total cholesterol (TC), triglycerides (TG), and low-density lipoprotein cholesterol (LDL-C) or decreased high-density lipoprotein cholesterol (HDL-C) levels [4]. The percentage of patients with PCOS treated with antilipids was however only 1.5% [5]. Dyslipidemia is an independent risk factor for cardiovascular disease (CVD), the occurrence of cardiovascular events increases the morbidity and mortality of patients with polycystic ovary syndrome. Effective control of blood lipid levels is also an important part of managing PCOS disease.

Vitamin D is a fat-soluble steroid, including animal-derived vitamin D_3_ and plant-derived vitamin D_2_. Vitamin *D* is synthesized by 25-hydroxylase in the body that synthesizes 25-hydroxy-vitamin D (25 (OH) D), which is the main storage form in the body, refl ecting the nutritional status of vitamin D in the body. 25 (OH) D becomes 1,25 dihydroxyvitamin D (1,25 (OH)_2_ D) by hydroxylation at the 1*α* position. It is the main active metabolite of vitamin D in the body, which binds to vitamin D receptors widely present in tissues to exert its effect [7]. Studies have found that the effect of vitamin D on blood lipids may be through the inhibition of PTH secretion as PTH had been proved to reduce lipolysis [8]. It can also increase the level of calcium to reduce the formation and secretion of TG in the liver [9]. In addition, vitamin D may also affect the secretion and sensitivity of insulin, thereby indirectly affecting blood lipids [10]. While vitamin D deficiency is very common in the general population, it is even more prevalent in patients with PCOS [11]. Considering the high prevalence of vitamin D deficiency in PCOS, vitamin D supplementation could be a simple and low-risk add-on to these therapies if its positive effects on lipid metabolic were proven to be true.

In recent years, there have been many published clinical randomized controlled trials (RCTs). Some studies have found that supplementation of a certain dose of vitamin D can reduce LDL-C levels and TC levels [12]. There have also been studies that found no significant difference in blood lipid levels between the two groups [13]. The conclusion is still controversial, so this article further systematically reviews existing studies to provide valuable evidence-based science for clinical work.

## 2. Materials and Methods

### 2.1. Search Strategy

This meta-analysis was performed in accordance with the Preferred Reporting Items for Systematic Reviews and Meta-Analysis (PRISMA) guideline [14]. Two authors (G. H and L-YT) independently searched PubMed, Embase, Cochrane Library, Web of Science, CNKI, and Wanfang databases until May 2020. A combination of subject words and free text terms was adopted to ensure the recall rate and precision rate. The search terms were as follows: polycystic ovary syndrome, PCOS, 25-hydroxy-vitamin D3, vitamin D, cholecalciferol, and calcitriol, and searched by logical operations.

### 2.2. Inclusion and Exclusion Criteria

Inclusion criteria were as follows: (1) patients: PCOS is diagnosed according to the 2003 Rotterdam criteria [15] or the 1990 National Institute of Child Health and Human Development criteria; (2) intervention: vitamin D, cholecalciferol, and calcitriol; (3) control: placebo; (4) outcome: total cholesterol, triglycerides, LDL-cholesterol, and HDL-cholesterol; (5) study design: randomized controlled trials.

Exclusion criteria were as follows: (1) review reports; (2) data is incomplete; (3) without relevant data; (4) nonrandom controlled trials; (5) repeat published articles.

### 2.3. Data Extraction

The two authors (L-YT和Y-WN) independently extracted information from the full text included, including the first author, year of publication, country, diagnosis, age, body mass index (BMI), sample size of the vitamin D group, sample size of the placebo group, medication course, medication dose, and reported outcome indicators.

### 2.4. Data Transformation

To convert the unit of blood lipid concentrations and accompanying variance measures from mmol/L to mg/dL, the values of TC, LDL-C, and HDL-C were multiplied by 39 and the triglyceride values by 88. Some studies reported TC and TG data using the median and the first and third quartiles. To be able to use these data in our meta-analysis, we converted them to mean and SD according to the method provided by Wan et al. [16] and Luo et al. [17]. This method is based on the assumption that the data are normally distributed, which we know is not the case. We decided to do a sensitivity analysis to check whether exclusion of these converted data would change the outcomes of our meta-analysis.

### 2.5. Quality Evaluation

The quality of the included studies was evaluated by Cochrane Collaboration's tool [18], including selection bias, performance bias, detection bias, attrition bias, reporting bias, and other types of bias. For each project, we assessed risk of bias as low, unclear, and high. Two authors (G. H和Y-WN) independently evaluated the quality of the literature and consulted the professor to resolve the dispute and reach an agreement.

### 2.6. Statistical Analysis

Using RevMan5.3 software for statistical analysis, the outcome indicators were expressed in WMD and 95% confidence intervals. For all statistical analyses, *P* value <0.05 was considered statistically significant. We used Cochrane's *Q* statistic and *I*^2^ statistic to judge the heterogeneity (*I*^2^ statistic >50% and *P* value <0.10 was used as a threshold indicating significant heterogeneity) [19]. The fixed-effects model was applied to the meta-analysis. If there was a significant heterogeneity between studies, a sensitivity analysis was conducted to assess the stability and heterogeneity of the included studies. A funnel plot was used to analyze the publication bias.

## 3. Results

### 3.1. Literature Search and Study Characteristics

**Figure 1:**
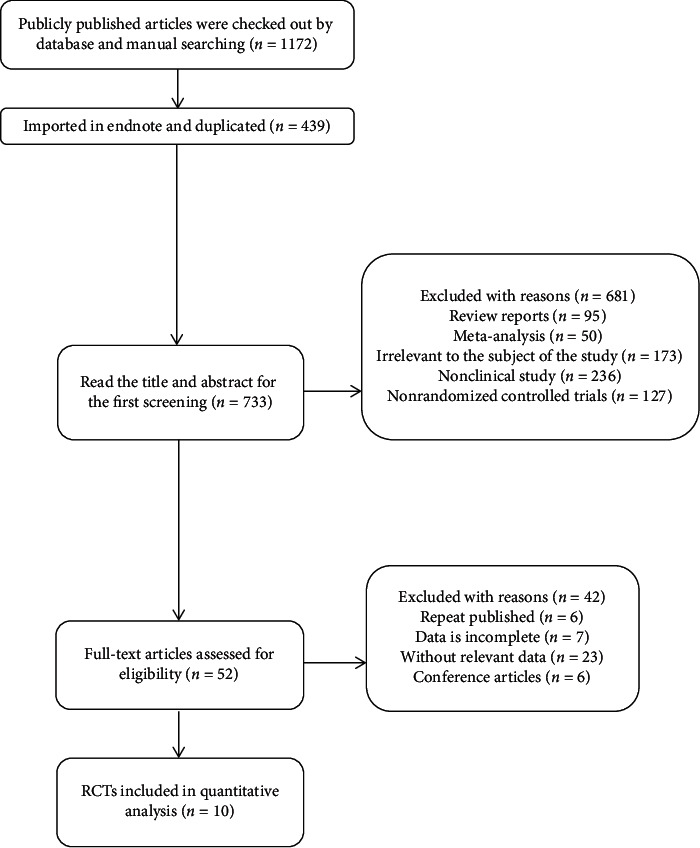
Flowchart of literature search.

Through searching Chinese and foreign databases, a total of 1172 articles were retrieved: 269 from PubMed, 320 articles from Embase, 179 articles from Cochrane Library, 216 articles from Web of Science, 125 articles from CNKI, and 63 articles from Wanfang. After importing the endnote to find duplicates, 439 articles were removed. According to the inclusion and exclusion criteria, 10 articles were finally included [12, 13, 20–27], including 543 subjects, all of which were in English. Retrieval flowchart and general characteristics of the included studies are shown in Figure 1 and Supplementary Table 1.

### 3.2. Quality Evaluation of Included Studies

**Figure 2:**
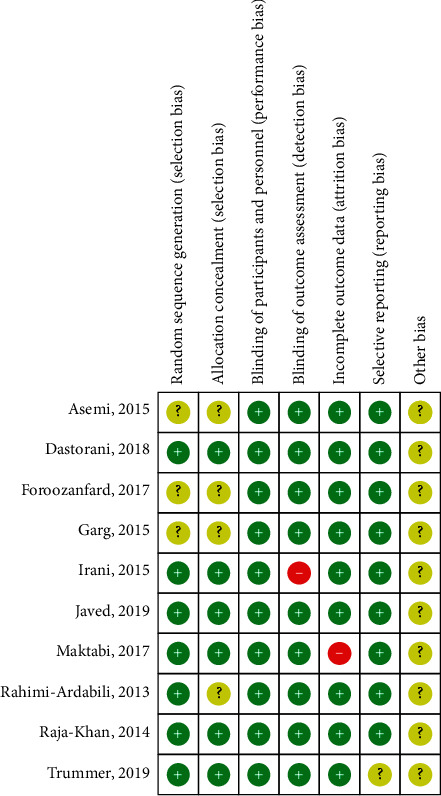
Risk of bias summary. (Note: The red with a minus means high risk of bias; the yellow with a question mark means unclear; the green with a plus means low risk of bias).

Three studies mentioned the word “random” and did not mention the specific random sequence generation method [22, 23, 25]. Seven studies used a computer random sequence generation method [12, 13, 20, 21, 24, 26, 27]. Four studies did not specifically describe the method of allocation concealment [20, 22, 23, 25], and six studies described in detail that there was no difference in size, color, or smell between the positive control group and the placebo at the time of allocation [12, 13, 21, 24, 26, 27]. Nine studies were double-blind and one study was single-blind [24]. One study did not specify the reasons for the loss of follow-up and withdrawal [26], and one study may have reporting bias due to the failure to list the outcome reports for LDL-C and HDL-C [27]; other biases are unknown (Figure 2).

### 3.3. Meta-Analysis Results

#### 3.3.1. Vitamin *D* Supplementation and Total Cholesterol

**Figure 3:**
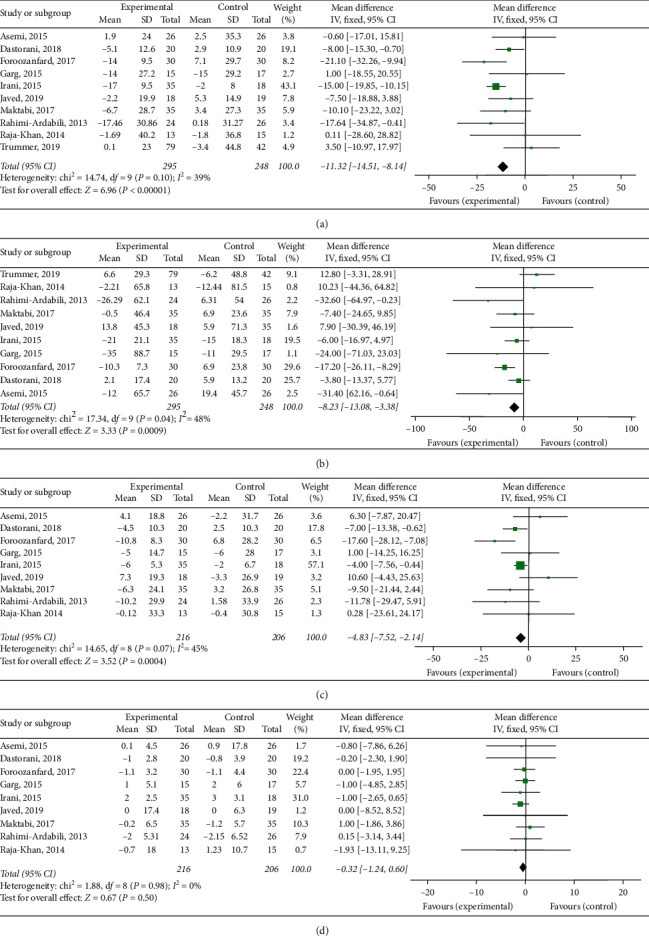
Forest plot presenting weighted mean difference and 95% confidence intervals (CIs) for the impact of vitamin D supplementation on TC level.

A total of 10 studies have reported the effect of vitamin D supplementation versus placebo on total cholesterol in patients with polycystic ovary syndrome. Heterogeneity test (*P*=0.10, *I*^2^ = 39%), so fixed-effect model was used, and the final results showed that vitamin D supplementation can reduce total cholesterol level (WMD = –11.32, 95%CI = [–14.51, -8.41], *P* < 0.00001) (Figure 3(a)).

#### 3.3.2. Vitamin *D* Supplementation and Triglyceride

A total of 10 studies have reported the effect of vitamin D supplementation versus placebo on triglyceride in patients with polycystic ovary syndrome. The heterogeneity test result is shown in Figure 3(b) (*P*=0.04, I2 = 48%), so a fixed-effect model was used, and the final results showed that vitamin D supplementation can reduce triglyceride level (WMD = –8.23, 95% CI = [–13.08, -3.38], *P*=0.0009) (Figure 3(b)).

#### 3.3.3. Vitamin *D* Supplementation and LDL-C

A total of nine studies have reported the effect of vitamin D supplementation versus placebo on LDL-C in patients with polycystic ovary syndrome. The heterogeneity test result is shown in Figure 3(c) (*P*=0.07, I2 = 45%), so a fixed-effect model was used, and the final results showed that vitamin D supplementation can reduce LDL-C level (WMD = –4.83, 95% CI = [–7.52, –2.14], *P*=0.0004) (Figure 3(c)).

#### 3.3.4. Vitamin *D* Supplementation and HDL-C

A total of nine studies have reported the effect of vitamin D supplementation versus placebo on HDL-C in patients with polycystic ovary syndrome. The heterogeneity test result is shown in Figure 3(d) (*P*=0.98, I2 = 0%), so a fixed-effect model was used, and the final results showed that there was no statistically significant difference in the effect of vitamin D supplementation on HDL-C levels (WMD = –0.32, 95% CI = [–1.24, 0.60], *P*=0.50) (Figure 3(d)).

### 3.4. Sensitivity Analysis

The “one by one elimination method” was used to find the source of heterogeneity. The sensitivity analysis for total cholesterol showed that the study of Trummer et al. [27] significantly affected the WMD. After excluding the study, the results still showed that vitamin D supplementation can reduce total cholesterol level in patients with polycystic ovary syndrome (WMD = –12.08, 95% CI = [–15.35, –8.81], *P* < 0.00001, I^2^ = 24%, *P* = 0.23). The sensitivity analysis for triglyceride showed that the study of Trummer et al. [27] significantly affected the WMD. After excluding the study, the results still showed that vitamin D supplementation can reduce triglyceride level in patients with polycystic ovary syndrome (WMD = –10.33,95% CI = [–15.41, –5.24], *P* < 0.001, I^2^ = 21%, *P*=0.26). The sensitivity analysis for LDL-C showed that the study of Foroozanfard et al. [25] significantly affected the WMD. After excluding the study, the results still showed that vitamin D supplementation can reduce LDL-C level in patients with polycystic ovary syndrome (WMD = –3.94, 95% CI = [–6.72, –1.16], *P*=0.006, I^2^ = 19%, *P*=0.28).

### 3.5. Publication Bias

**Figure 4:**
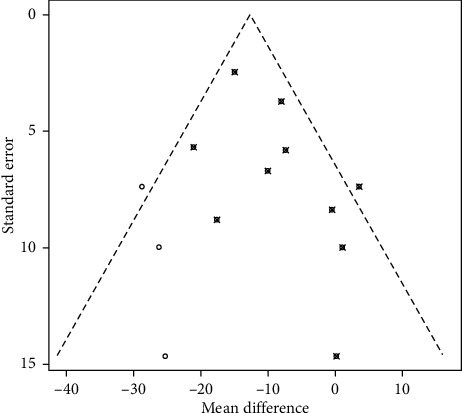
Funnel plot for the impact of vitamin D supplementation on total cholesterol levels after trim-and-fill analysis. (Note: ○ represent newly included studies, ⦻ represent originally included studies).

**Figure 5:**
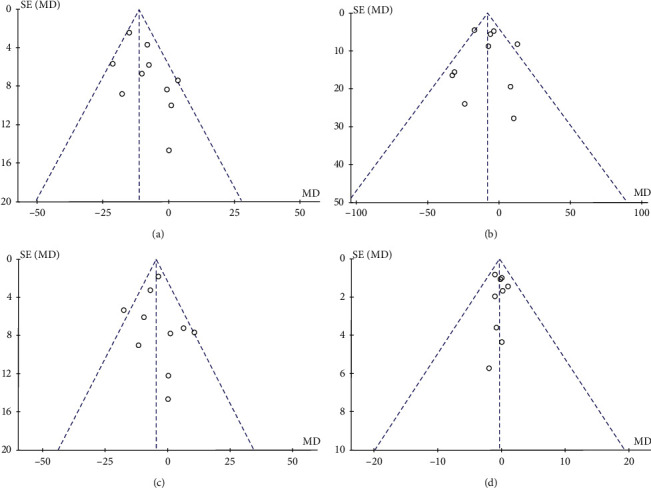
(a). Funnel plot for the impact of vitamin D supplementation on total cholesterol levels. (b). Funnel plot for the impact of vitamin D supplementation on triglycerides levels. (c). Funnel plot for the impact of vitamin D supplementation on LDL-C levels. (d). Funnel plot for the impact of vitamin D supplementation on HDL-C level.

The funnel plot was drawn by four outcome indicators. The funnel plot of the total cholesterol outcome was asymmetrically distributed, with publication bias, which may be related to unpublished negative results. After trim-and-fill analysis was used [28], it can be seen from Figure 4 that, to eliminate publication bias, three studies need to be included. In addition, the effect size after clipping was WMD = –12.63, 95% CI = [–15.68; –9.57], *P* < 0.0001, which was not much different from the original effect size of WMD = –11.32, 95% CI = [–14.51, –8.41], *P* < 0.00001, indicating that the existence of mild publication bias had no substantial effect on the overall results. The funnel plot for the remaining three outcomes was basically symmetrical, and it was considered that the three outcome indicators were less likely to be biased and the conclusion was more reliable. The funnel plots are shown in Figure 5.

## 4. Discussion

This meta-analysis of 10 RCTs evaluating the effect of vitamin D supplementation on lipids revealed that vitamin D supplementation has a beneficial effect on serum TC, LDL-C, and TG but not on HDL-C. This is a study on patients with PCOS, which provides another option for adjusting blood lipids for PCOS patients with dyslipidemia. Dibaba et al. [29] analyzed 41 RCTs, and it was reported that vitamin D supplementation can reduce TC, TG, and LDL-C, but it is not beneficial for HDL-C. In research subjects with vitamin D deficiency (VDD) especially, the effect is more obvious. Our results are consistent with the results of this study in a broad population. Pergialiotis et al. [30] included two RCTs from 81 patients with polycystic ovary syndrome and found that vitamin D supplementation is effective for TC but not for LDL-C. The reason may be that the sample size is small and the statistical effect is not achieved, which does not mean that there is no effect. Xue et al. [31] included two single-arm studies and four RCTs containing 156 subjects with polycystic ovary syndrome. Vitamin *D* supplementation showed a statistical significance in terms of reducing TG, but there was no statistical significance in terms of reducing LDL-C. Because single-arm clinical trials cannot be randomized and blinded, the strength of the argument is slightly weaker, and the conclusions are unreliable. Miao et al. [32] combined six RCTs and showed that vitamin D intervention reduced serum TC and LDL-C levels, while TG and HDL-C levels remained unchanged. However, the baseline data has a greater consistency, and the final data are directly merged, resulting in unstable results.

Our study is a meta-analysis with the largest number of research objects, a relatively wide area, and a more reliable and stable outcome. Incorporating a large number of research objects can greatly reduce sampling errors, increase the reliability of conclusions, and improve inspection efficiency. Moreover, the research subjects come from Europe, Asia, and North America, which reduces the regional selection bias and is representative. All included studies are high-quality RCTs, and the outcome indicators are analyzed using baseline change values, which reduces the heterogeneity of baseline data to a certain extent. The sensitivity analysis of TC, TG, and LDL-C outcome indicators was carried out, respectively. In the sensitivity analysis of the TC and TG outcome indicators, the article by Trummer et al. based on the research data expressed in the interquartile range was excluded, and it was found that the heterogeneity had decreased, but the treatment results were still effective. Because the research data of Trummer et al. are skewed, it contributes a certain degree of heterogeneity. And after eliminating other studies one by one, there was no impact on heterogeneity and treatment results. In the sensitivity analysis of the LDL-C outcome indicators, it was finally found that Foroozanfard et al.'s article was excluded, and it was found that the heterogeneity decreased, but it did not affect the final result. As the general information of the subjects in the Foroozanfard article is not fully reported, the unknown age and BMI may be the source of heterogeneity Because the meta-analysis indicated an evidence of publication bias for TC, trim-and-fill analysis was adopted. Three studies need to be included after the trim-and-fill analysis to eliminate publication bias. Further analysis found that vitamin D supplementation is still beneficial to total cholesterol, indicating that mild publication bias did not affect the results. No publication bias was found for the remaining three indicators, indicating that the outcome indicators are reliable. Once again, the four outcome indicators studied are stable and reliable.

It is well known that metabolic syndrome and VDD are common in PCOS. At present, studies have found that the mechanism of vitamin D's effect on lipids mainly includes the following aspects: Li et al. [33] found that, in the state of vitamin D deficiency, VDR activity is significantly reduced, and it will lead to an increase in circulating cholesterol levels through Insig-2/SREBP-2/HMGR-dependent signaling pathways. Intervention with active vitamin D3 can significantly inhibit the increase in cholesterol synthesis caused by VD deficiency through restoring VDR transcriptional activity and Insig-2 expression. Koszowska et al. [34] found that 1.25 (OH) 2D3 can reduce the triglyceride content in liver cells and inhibit the proliferation and differentiation of preadipocytes (3T3-Ll cells). Simultaneously, it can also affect the expression of the enzymes related to lipid metabolism G3PDH, ACC1, ACS1, HSL, adipokines APN, and visfatin mRNA. A multicenter study conducted by Browne et al. [35] found that 1,25 (OH) 2D3 increased the level of Apo AI (the major constituent of HDL-C) and decreased the level of Apo B (the major constituent of LDL-C). However, it has been documented that VDR activation suppresses apolipoprotein AI (Apo AI) gene expression [18, 36]. Therefore, the effect of vitamin D on HDL-C is unclear. Studies have found that oxidative stress is closely related to polycystic ovary syndrome and increases the concentration of lipid peroxides [37, 38]. The systematic review and meta-analysis published by Akbari et al. [39] demonstrated that vitamin D supplementation can help improve the levels of inflammatory indicators hs-CRP and oxidative stress indicators MDA and TAC in patients with PCOS but did not affect NO and GSH levels. It still shows that vitamin D supplementation has a certain antioxidant effect on patients with PCOS, which may improve blood lipid levels. Perhaps, vitamin D deficiency is not simply a manifestation of PCOS, it is very likely that vitamin D deficiency is related to the pathogenesis of PCOS. So, supplementing a certain dose of vitamin D seems to be a beneficial choice for PCOS patients.

The study has several limitations. The first point is that the follow-up period is too short to further evaluate the safety of vitamin D supplementation. During the process of vitamin D supplementation, attention should be paid to monitoring serum calcium and phosphorus levels and renal function. Beware of hypercalcemia and renal insufficiency caused by excessive vitamin D. The second point is that the influence of latitude, season, and other factors on vitamin D is not considered. These factors affect the absorption of vitamin D. The third point is the failure to further clarify the reasonable dosage range of vitamin D supplementation. The dose included in this study is 50000 IU/20 days and12000 IU/day. The dose is different, and there is no further guidance on the amount of vitamin D in the clinical treatment of PCOS.

In conclusion, this meta-analysis shows that vitamin D supplementation can improve the blood lipid profile of patients with polycystic ovary syndrome, but it fails to improve the high-density lipoprotein level. There are still shortcomings in the current research, and we look forward to multicenter, large-sample randomized controlled trials in the future to further determine the optimal dose of vitamin D supplementation for patients with polycystic ovary syndrome who have high-risk cardiovascular events.

## Data Availability

The data used in the study are available upon request to the corresponding author.

## Supplementary Materials

Supplementary Materials Table 1: baseline characteristics of included studies.
