# Supplementary material for: The Effect of Vitamin D Supplementation on Blood Lipids in Patients with Polycystic Ovary Syndrome: A Meta-Analysis of Randomized Controlled Trials
Source: Int J Endocrinol. 2021 Jan 30;2021:8849688. doi: 10.1155/2021/8849688 (PMC7868162; doi:10.1155/2021/8849688)
Supplement: Supplementary Materials — Table 1: baseline characteristics of included studies. [file 8849688.f1.docx]

Raw data collected：mg/dl

| StudyID | TC  Vit D | placeo | TG  V | P | LDL  V | P | HDL  V | P |
| --- | --- | --- | --- | --- | --- | --- | --- | --- |
| Rahimi-Ardabili | -17.46±30.86 | 0.18±31.27 | -26.29±62.1 | 6.31±54.0 | -10.20±29.9 | 1.58±33.9 | -2.00±5.31 | -2.15±6.52 |
| Raja-Khan | -1.69±40.2 | -1.80±36.8 | -2.21±65.8 | -12.44±81.5 | -0.12±33.3 | -0.40±30.8 | -0.70±18.0 | 1.23±10.7 |
| Asemi | 1.9±24.0 | 2.5± 35.3 | -12.0± 65.7 | 19.4 ± 45.7 | 4.1± 18.8 | -2.2± 31.7 | 0.1± 4.5 | 0.9± 17.8 |
| Garg | -14.0±27.2 | -15.0±29.2 | -35.0±88.7 | -11.0±29.5 | -5.0±14.7 | -6.0±28.0 | 1.0±5.1 | 2.0±6.0 |
| Irani | -17.0±9.5 | -2.0±8.0 | -21.0±21.1 | -15.0±18.3 | -6.0±5.3 | -2.0±6.7 | 2.0±2.5 | 3.0±3.1 |
| Foroozanfard | –14.0 ± 9.5 | 7.1± 29.7 | -10.3 ± 7.3 | 6.9 ± 23.8 | -10.8 ± 8.3 | 6.8 ± 28.2 | –1.1 ± 3.2 | –1.1 ± 4.4 |
| Maktabi | −6.7±28.7 | 3.4±27.3 | −0.5±46.4 | 6.9±23.6 | −6.3±24.1 | 3.2±26.8 | −0.2±6.5 | −1.2±5.7 |
| Dastorani | −5.1± 12.6 | 2.9± 10.9 | 2.1± 17.4 | 5.9± 13.2 | −4.5± 10.3 | 2.5± 10.6 | −1.0± 2.8 | −0.8 ± 3.9 |
| Javed | -2.2±19.9 | 5.3±14.9 | 13.8±45.3 | 5.9±71.3 | 7.3±19.3 | -3.3±26.9 | 0.0±17.4 | 0.0±6.3 |
| Trummer | 0.1±23.0 | -3.4±44.8 | 6.6±29.3 | -6.2±48.8 |  |  |  |  |
|  |  |  |  |  |  |  |  |  |

Note：Total cholesterol Triglycerides LDL-cholesterol HDL-cholesterol

V-Vit

P-placeo
